# *OsPIN1b* is Involved in Rice Seminal Root Elongation by Regulating Root Apical Meristem Activity in Response to Low Nitrogen and Phosphate

**DOI:** 10.1038/s41598-018-29784-x

**Published:** 2018-08-29

**Authors:** Huwei Sun, Jinyuan Tao, Yang Bi, Mengmeng Hou, Jiajing Lou, Xinni Chen, Xuhong Zhang, Le Luo, Xiaonan Xie, Koichi Yoneyama, Quanzhi Zhao, Guohua Xu, Yali Zhang

**Affiliations:** 1 State Key Laboratory of Crop Genetics and Germplasm Enhancement, and Key Laboratory of Plant Nutrition and Fertilization in Low-Middle Reaches of the Yangtze River, Ministry of Agriculture, Nanjing Agricultural University, Nanjing, 210095 China; 2 College of Agronomy, Collaborative Innovation Center of Henan Grain Crops, Key Laboratory of Rice Biology in Henan Province, Henan Agricultural University, Zhengzhou, 450002 China; 3 Center for Bioscience Research & Education, Utsunomiya University, 350 Mine-machi, Utsunomiya, 321-8505 Japan

## Abstract

The response of plant root development to nutrient deficiencies is critical for crop production. Auxin, nitric oxide (NO), and strigolactones (SLs) are important regulators of root growth under low-nitrogen and -phosphate (LN and LP) conditions. Polar auxin transport in plants, which is mainly dependent on auxin efflux protein PINs, creates local auxin maxima to form the basis for root initiation and elongation; however, the *PIN* genes that play an important role in LN- and LP-modulated root growth remain unclear. qRT-PCR analysis of *OsPIN* family genes showed that the expression of *OsPIN1b* is most abundant in root tip and is significantly downregulated by LN, LP, sodium nitroprusside (SNP, NO donor), and GR24 (analogue of SLs) treatments. Seminal roots in *ospin1b* mutants were shorter than those of the wild type; and the seminal root, [^3^H]IAA transport, and IAA concentration responses to LN, LP, SNP, and GR24 application were attenuated in *ospin1b-1* mutants. *pCYCB1;1::GUS* expression was upregulated by LN, LP, SNP, and GR24 treatments in wild type, but not in the *ospin1b-1* mutant, suggesting that *OsPIN1b* is involved in auxin transport and acts as a downstream mediator of NO and SLs to induce meristem activity in root tip in rice under LN and LP.

## Introduction

Nitrogen (N) and phosphate (P) are required for plant growth and development; however, the natural supply of soil N and P limits plant growth and development in most agricultural cropping systems^1,2^. Therefore, the response of the root system architecture to N and P deficiencies is critical for plant growth and crop productivity. Increases in the root-to-shoot ratio and root surface area regulated by deficiencies of N and P have been studied in several plant species^3–5^, most of which focused on the responses of the root system to P deficiency. Two contrasting reactions of root growth to P deficiency have been recorded: 1) reduction of primary root growth and enhancement of lateral root (LR) density in *Arabidopsis*^3,6–9^; and 2) elongation of primary roots in other plant species (such as rice)^10,11^, suggesting that P deficiency-related changes in the root morphology are complex and vary according to the experimental conditions and plant species. Moreover, in maize and rice, elongation of primary roots and inhibition of LR are typical responses to N deprivation^4,12–14^.

Root architecture is a highly plastic trait, and its plasticity is strongly controlled by environmental conditions and plant regulators. Polar auxin transport and auxin signaling pathways play essential roles in root development^15–17^. Polar auxin transport in plants creates local auxin maxima that form the basis for root initiation and elongation^18–20^. Polar auxin transport is mainly mediated by auxin efflux carrier PIN family proteins^21^. Some lines of evidence suggest that the *OsPIN* family genes participate in root formation and elongation in rice^22–25^. Moreover, the inhibited auxin transport from the shoot to root, which is largely dependent on reduced expression of *OsPIN* genes, plays an important role in low nitrogen (LN)- and low phosphate (LP)-modulated LR formation and seminal root elongation in rice^12^. Few studies have assessed the *PIN* genes involved in auxin polar transport and LN- and LP-mediated modulation of root development.

Root development involves cross-talk among several plant hormones. Auxins play an important role in regulating root growth and development under low N and low P conditions^12^. In addition to auxin, strigolactones (SLs) and nitric oxide (NO) are associated with the regulation of root development^12,26–42^. Several lines of evidence suggest that the SL pathway is involved in rice root elongation under LN and LP conditions^12,34^. NO, as a signaling molecule, plays a pivotal role in root growth modulation^43^. NO participates in LN- and LP-induced seminal root elongation in rice^43^. SL signaling genes are required for NO-induced elongation of seminal root in response to LN and LP in rice plants^43^. Moreover, SLs are believed to modulate auxin transport to regulate root formation and elongation^12,32,33^. Although NO is closely associated with auxin by regulating *PIN* family genes and root elongation in plants^30^, whether auxin transport plays a role in NO-modulated root elongation under LN- and LP-deficient conditions remains unclear.

In this study, phenotypic, cellular, and genetic analyses of rice were performed to explore the role of the *OsPIN* gene in regulating auxin polar transport and root development, and the crosstalk between SLs and NO under LN and LP conditions. We found that SLs are required for NO-mediated modulation of LN- and LP-induced seminal root elongation (similar responses of seminal and adventitious roots to LN and LP) and function by inhibiting auxin transport to the root tip. Of 10 *OsPINs*, the expression of *OsPIN1b*, which encodes an auxin efflux carrier, was highest in rice root tip, and so might be involved in auxin transport and elongation of seminal root by regulating meristem activity in the root tips of rice plants under LN and LP conditions.

## Results

### NO regulates root elongation by inhibiting polar auxin transport

LN- and LP-enhanced NO production resulted in the elongation of seminal roots (Supplementary Fig. S1a,c,d), as reported previously^44^. The effects of NO on auxin status in rice were investigated using transgenic plants expressing the *pDR5::GUS* construct. Compared with the control, *pDR5::GUS* intensity and IAA concentrations decreased in LN- and LP-treated root tips of WT plants (Supplementary Fig. S1b–e). The application of sodium nitroprusside (SNP, NO donor) under normal nutrition conditions decreased *pDR5::GUS* expression and IAA concentration in root tips to a level similar as that under nutrient-deficient conditions. Application of cPTIO (2-(4-carboxyphenyl)-4,4,5,5- tetramethylimidazoline-1-oxyl-3-oxide, NO scavenger) under low-nutrient conditions markedly increased *pDR5::GUS* expression and IAA concentration in root tips to a level similar to that under control conditions (Supplementary Fig. S1b–e). However, *pDR5::GUS* expression and seminal root length showed no differences between control and control + cPTIIO (Fig. S1f–h), suggesting that the LN- and LP-mediated increase in NO levels reduced auxin levels in rice root tips.

**Figure 1:**
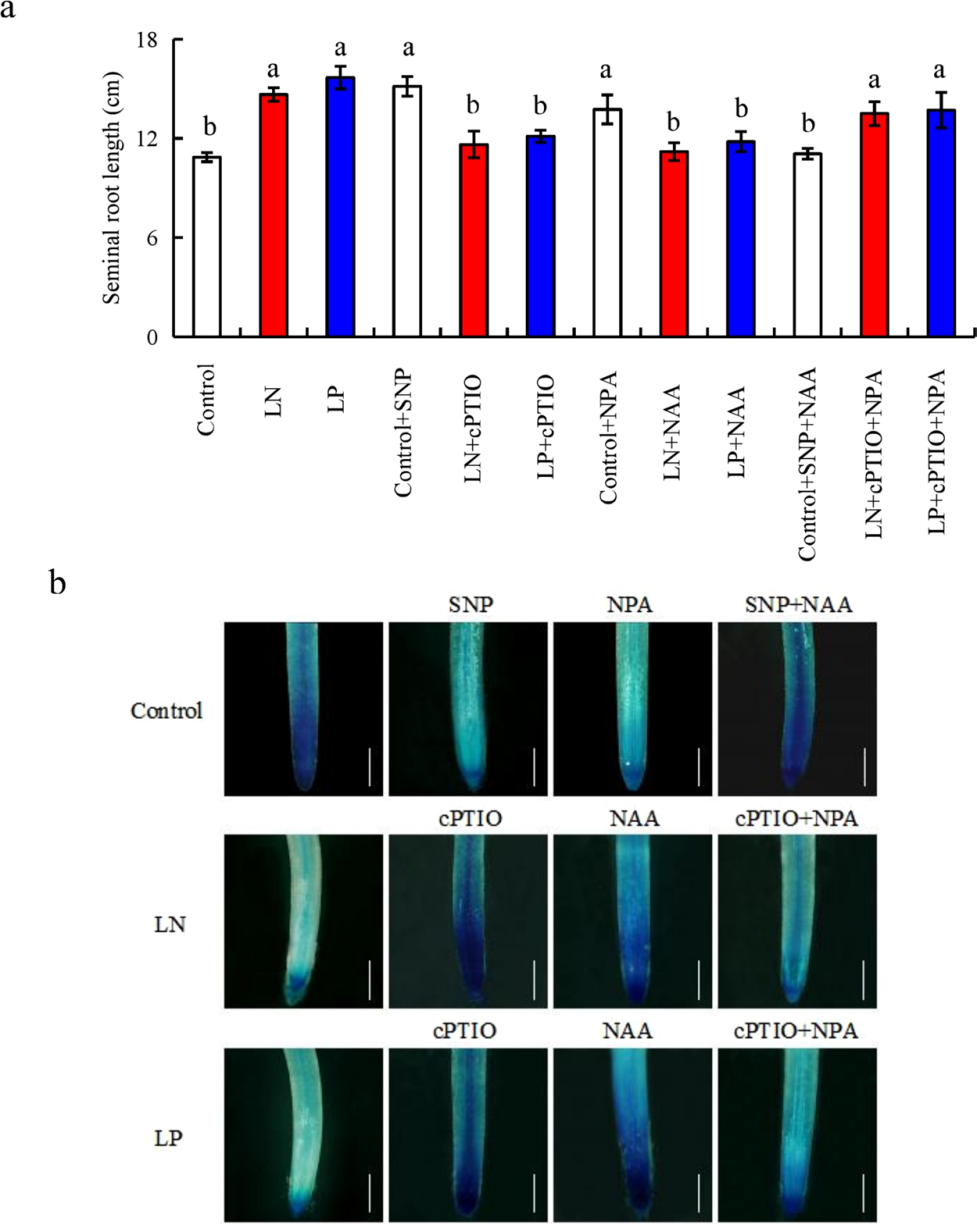
The seminal root length and histochemical localization of *pDR5::GUS* expression in the root tip of wild-type (WT) rice seedlings (Shiokari). Seedlings were grown in hydroponic medium containing normal nutrition (control; 2.5 mM N, 300 μM P), low N, or low P (LN, 0.02 mM; LP, 2 µM), or subjected to treatment with sodium nitroprusside (SNP, 10 µM), 2-(4-carboxyphenyl)-4,4,5,5- tetramethylimidazoline-1-oxyl-3-oxide (cPTIO, 80 µM), or a-naphthylacetic acid (NAA, 10 nM), or localized application of N-1-naphthylphthalamic acid (NPA, 20 µM) by dispensing diluted agar onto the root-shoot junction for 14 days. (**a**) Seminal root length; (**b**) histochemical localization of *pDR5::GUS* expression in root tip. Bar = 1 mm. Data are means ± SE of eight replicates and bars with different letters indicate significant differences at p < 0.05, as determined by ANOVA followed by the LSD test.

As shown in Fig. 1a,b, the application of naphthylacetic acid (NAA; an analogue of IAA) under LN and LP conditions reduced seminal root length and increased *pDR5::GUS* expression to levels similar to those under normal nutrition conditions. Moreover, application of NAA and SNP under control conditions markedly relieved the inductive effect of SNP application on seminal root elongation and its inhibitory effect on *pDR5::GUS* expression. Therefore, NO modulates LN- and LP-induced seminal root elongation by reducing auxin levels. In rice plants treated with N-1-naphthylphthalamic acid (NPA, a polar auxin transport inhibitor) under control conditions, the length of seminal roots markedly increased and the *pDR5::GUS* expression in the root tip significantly decreased to levels similar to those under low-nutrient conditions. Furthermore, application of NPA and cPTIO under LN and LP conditions suppressed the inhibitory effect of cPTIO [2-(4-carboxyphenyl)-4,4,5,5-tetramethylimidazoline-1-oxyl-3-oxide, an scavenger of NO] on seminal root elongation and its inductive effect on *pDR5::GUS* expression. These results suggested that NO negatively regulates auxin polar transport and reduces auxin levels in the root tip.

**Figure 2:**
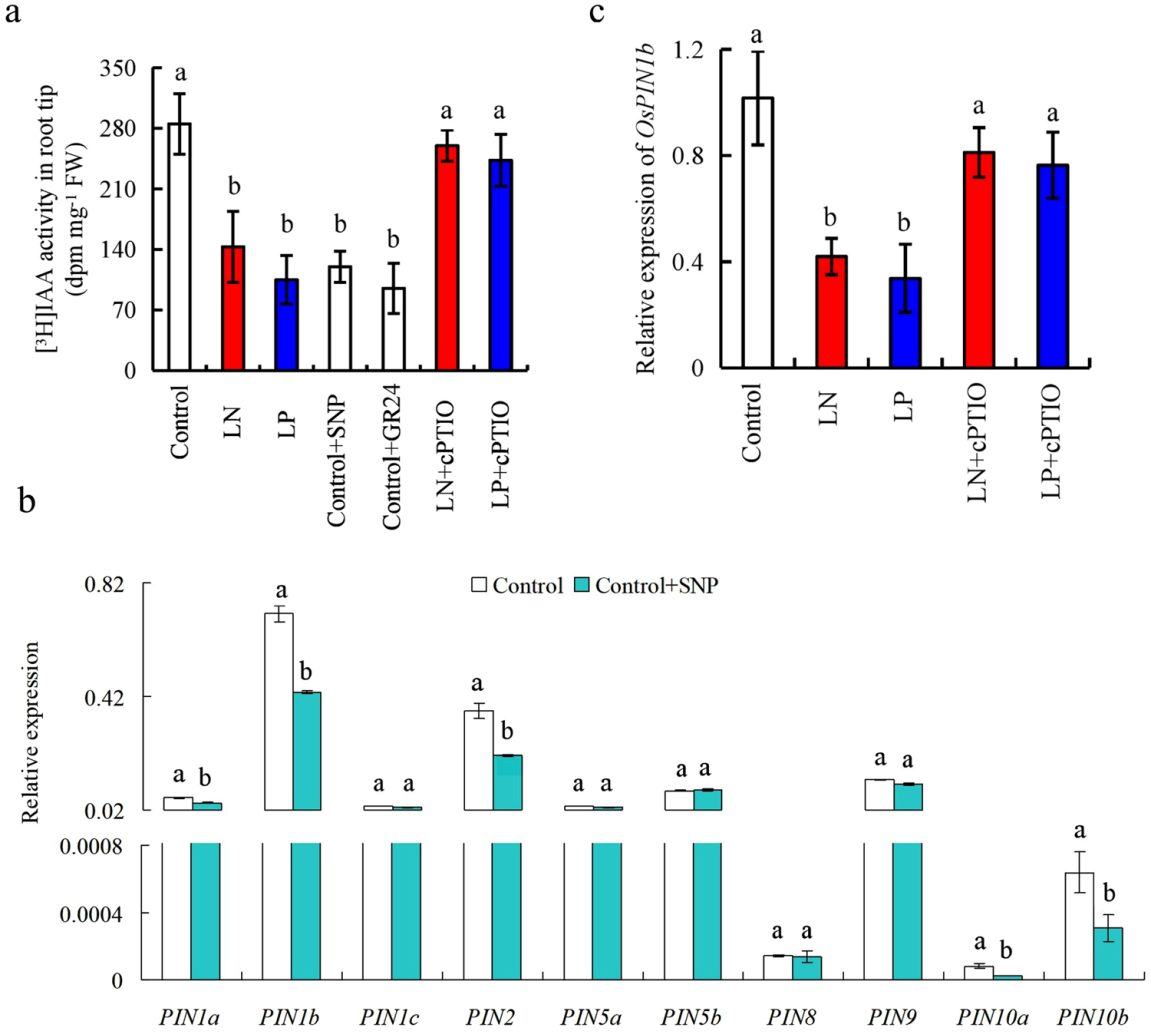
[^3^H]IAA transport and *PIN* family expression in root of WT rice seedlings (Shiokari). Seedlings were grown in hydroponic medium containing normal nutrition (control; 2.5 mM N, 300 μM P), low N, or low P (LN, 0.02 mM; LP, 2 µM P), or subjected to treatment with sodium nitroprusside (SNP, 10 µM), 2-(4-carboxyphenyl)-4,4,5,5- tetramethylimidazoline-1-oxyl-3-oxide (cPTIO, 80 µM), or GR24 (analog of SLs, 2.5 µM) for 14 days. (**a**) [^3^H]IAA transport; (**b**) *PIN* family expression; and (**c**), Os*PIN1b* expression. Data are means ± SE of eight replicates (**a**) and three replicates (**b**,**c**). Bars with different letters indicate significant differences at p < 0.05, as determined by ANOVA followed by the LSD test.

We evaluated the effects of SNP, cPTIO, and GR24 (an analogue of SLs) on [^3^H]IAA transport (Fig. 2a). [^3^H]IAA activity was lower in LN- and LP-treated root tips than in the control, suggesting that LN and LP decreased [^3^H]IAA transport from shoots to roots. Moreover, application of SNP and GR24 under control conditions markedly reduced [^3^H]IAA transport to root tips to the same extent as LP and LN; conversely, treatment with cPTIO under LN and LP conditions markedly increased [^3^H]IAA transport to root tips to the same extent as under control conditions (Fig. 2a). Quantitative reverse transcription polymerase chain reaction (qRT-PCR) analysis showed that *OsPIN1a-b*, *OsPIN2*, and *OsPIN10a-b* expression levels in WT root were significantly reduced by SNP treatment compared with those in the control (Fig. 2b). *OsPIN1b* expression was downregulated by LN and LP and restored by cPTIO in WT roots (Fig. 2c). These findings confirmed that NO modulates LN- and LP-induced seminal root elongation by suppressing polar auxin transport.

### Strigolactones and strigolactone signaling are required for NO-mediated suppression of polar auxin transport

**Figure 3:**
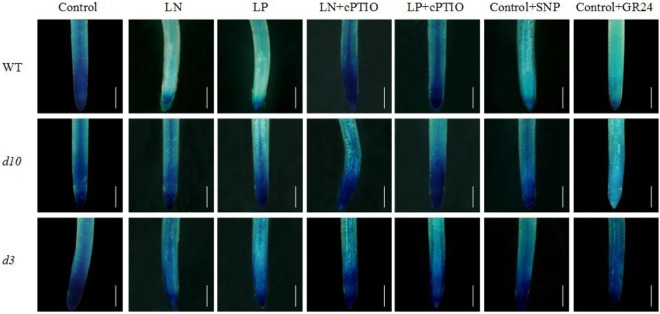
Histochemical localization of *pDR5::GUS* expression in root tip. Seedlings were grown in hydroponic medium containing normal nutrition (control; 2.5 mM N, 300 μM P), low N, or low P (LN, 0.02 mM; LP, 2 µM), or subjected to treatment with sodium nitroprusside (SNP, 10 µM), 2-(4-carboxyphenyl)-4,4,5,5-tetramethylimidazoline-1-oxyl-3-oxide (cPTIO, 80 µM), or GR24 (analog of SLs, 2.5 µM) for 14 days. Bar = 1 mm; n = 8.

**Figure 4:**
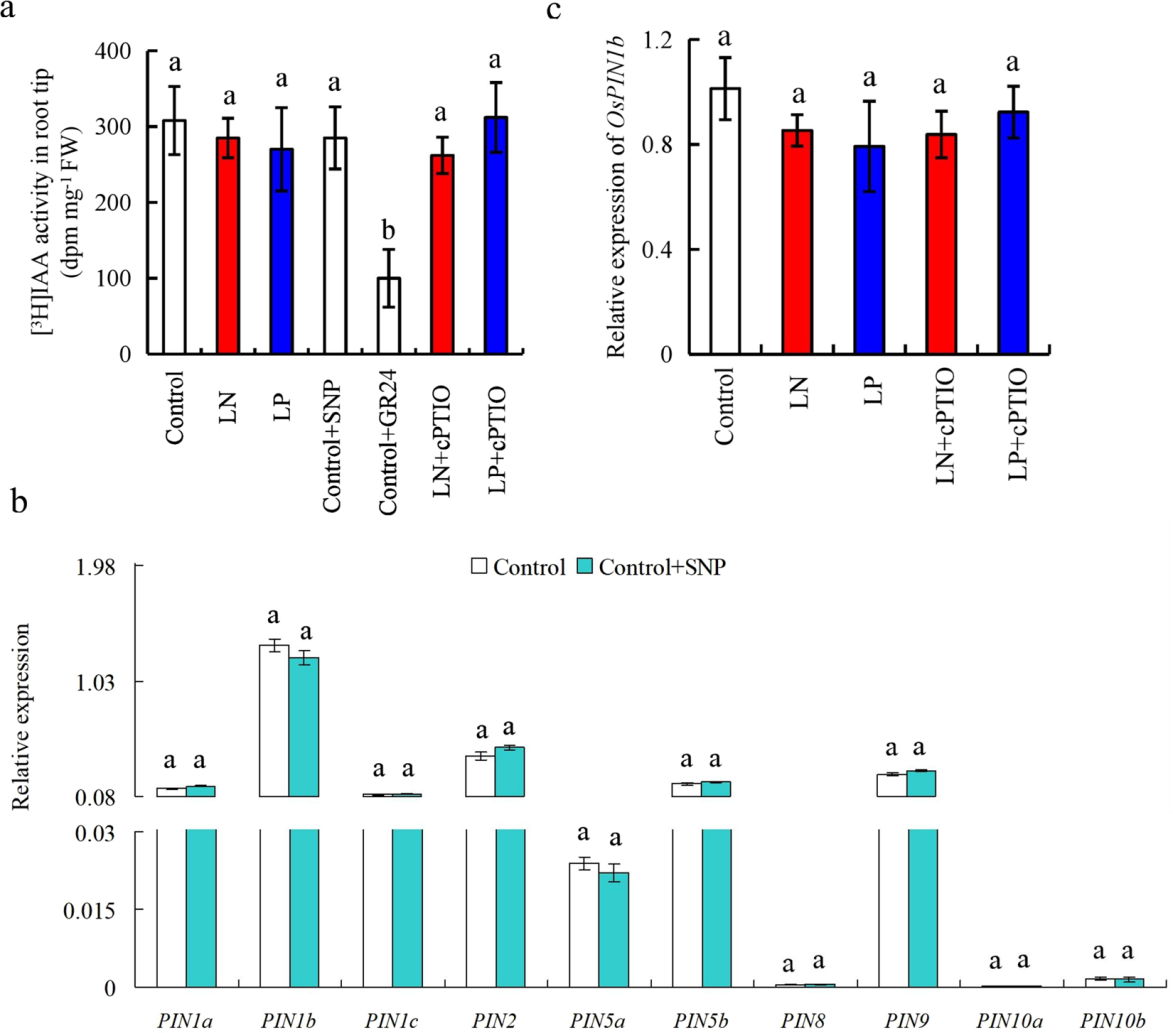
[^3^H]IAA transport and *PIN* family expression in root of the *d10* mutant. Seedlings were grown in hydroponic medium containing normal nutrition (control; 2.5 mM N, 300 μM P), low N, or low P (LN, 0.02 mM; LP, 2 µM) in addition to sodium nitroprusside (SNP, 10 µM), 2-(4-carboxyphenyl)-4,4,5,5-tetramethylimidazoline-1-oxyl-3-oxide (cPTIO, 80 µM), or GR24 (analog of SLs, 2.5 µM) for 14 days. (**a**) [^3^H]IAA transport; (**b**) *PIN* family expression; and (**c**) Os*PIN1b* expression. Data are means ± SE of eight replicates (**a**) and three replicates (**b**,**c**). Data are means ± SE of eight replicates and bars with different letters indicate significant differences at p < 0.05, as determined by ANOVA followed by the LSD test.

SLs are required for the role of NO in LN- and LP-induced seminal root elongation^43^. Under control conditions, application of SNP or GR24 reduced *pDR5::GUS* expression in WT root tips (Fig. 3). However, LN and LP treatments and application of SNP did not affect *pDR5::GUS* expression in SL synthetic and signaling mutants (*d10* and *d3*). Compared with control conditions, no change in [^3^H]IAA transport from shoots to root tips was observed in the *d10* mutant under low-nutrient conditions with the application of cPTIO (Fig. 4a). However, application of GR24 under control conditions markedly reduced [^3^H]IAA transport to root tips (Fig. 4a). No difference in the expression of *OsPIN* family genes was observed in the roots of the *d10* mutant between control and SNP conditions (Fig. 4b, Supplementary Fig. S2). In comparison to control conditions, *OsPIN1b* expression in the *d10* mutant was unchanged under LN, LP, and cPTIO treatments (Fig. 4c). These results confirmed that SLs and SL signaling are required for NO-mediated suppression of polar auxin transport in rice plants under LN and LP conditions.

### *OsPIN1b* is involved in NO- or SL-induced root elongation under LN and LP conditions

**Figure 5:**
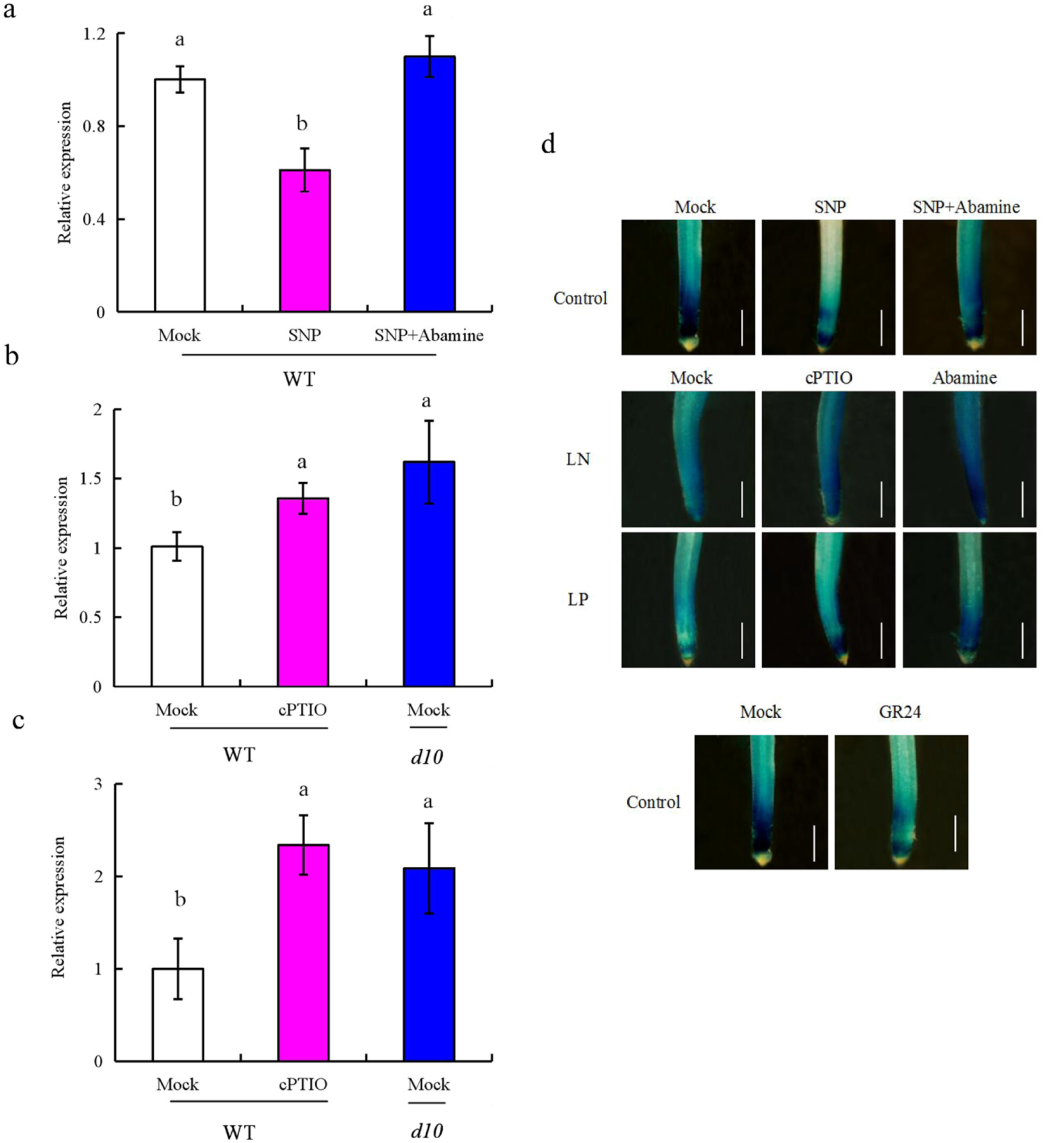
*OsPIN1b* expression and localization of *pPIN1b::GUS* expression in root tip. Seedlings were grown in hydroponic medium containing normal nutrition (control; 2.5 mM N, 300 μM P), low N, or low P (LN, 0.02 mM; LP, 2 µM ), or subjected to treatment with sodium nitroprusside (SNP, 10 µM), 2-(4-carboxyphenyl)-4,4,5,5- tetramethylimidazoline-1-oxyl-3-oxide (cPTIO, 80 µM), GR24 (analog of SLs, 2.5 µM), or abamine (100 µM) for 14 days. (**a**–**c**) *OsPIN1b* expression under control (**a**), LN (**b**), and LP (**c**) conditions. (**d**) Localization of *pPIN1b::GUS* expression. mRNA levels were normalized to that of *OsACT*. Bar = 1 mm. Data are means ± SE of three replicates and bars with different letters indicate significant differences at p < 0.05, as determined by ANOVA followed by the LSD test.

We analysed 10 *OsPIN* family genes in root tip of rice plants (Supplementary Fig. S3). Relative expression of *OsPIN1b* was the highest of the *OsPINs* in rice root tip, consistent with a previous study of other rice cultivars^44^. Therefore, *OsPIN1b* was used as the target gene in subsequent analyses. Compared with control conditions, *OsPIN1b* expression in root tip was significantly reduced by SNP treatment; however, application of abamine (an SL inhibitor) ameliorated this effect (Fig. 5a). Moreover, *OsPIN1b* expression was significantly upregulated by cPTIO application under LN or LP conditions in the WT compared with mock treatments, to a level similar to that in the *d10* mutant under LN and LP conditions (Fig. 5b,c).

Next, *GUS* expression driven by the *OsPIN1b* promoter in root tip was evaluated (Fig. 5d). Compared with control conditions, *pPIN1b::GUS* expression in root tip was significantly reduced by SNP and GR24 treatments; however, the application of abamine ameliorated this effect. Moreover, under LN and LP conditions, the expression of *pPIN1b::GUS* was significantly upregulated by the application of cPTIO or abamine in comparison with that in the mock treatments, consistent with the gene expression results. Therefore, *OsPIN1b* is involved in the NO- and SL-induced elongation of seminal root under LN and LP conditions.

### *ospin1b* mutants are less sensitive to LN and LP conditions, and SNP and GR24 treatments

The effect of LN and LP conditions on seminal root length of *ospin1b-1* and *ospin1b-2* and the WT (Dongjin) was assessed. Molecular characterisation revealed that T-DNAs were inserted into the promoter and 5′-URT regions, respectively, of the two *ospin1b* mutants. Compared with WT plants, *OsPIN1b* expression was almost completely suppressed in both *ospin1b* lines (Supplementary Fig. S4).

**Figure 6:**
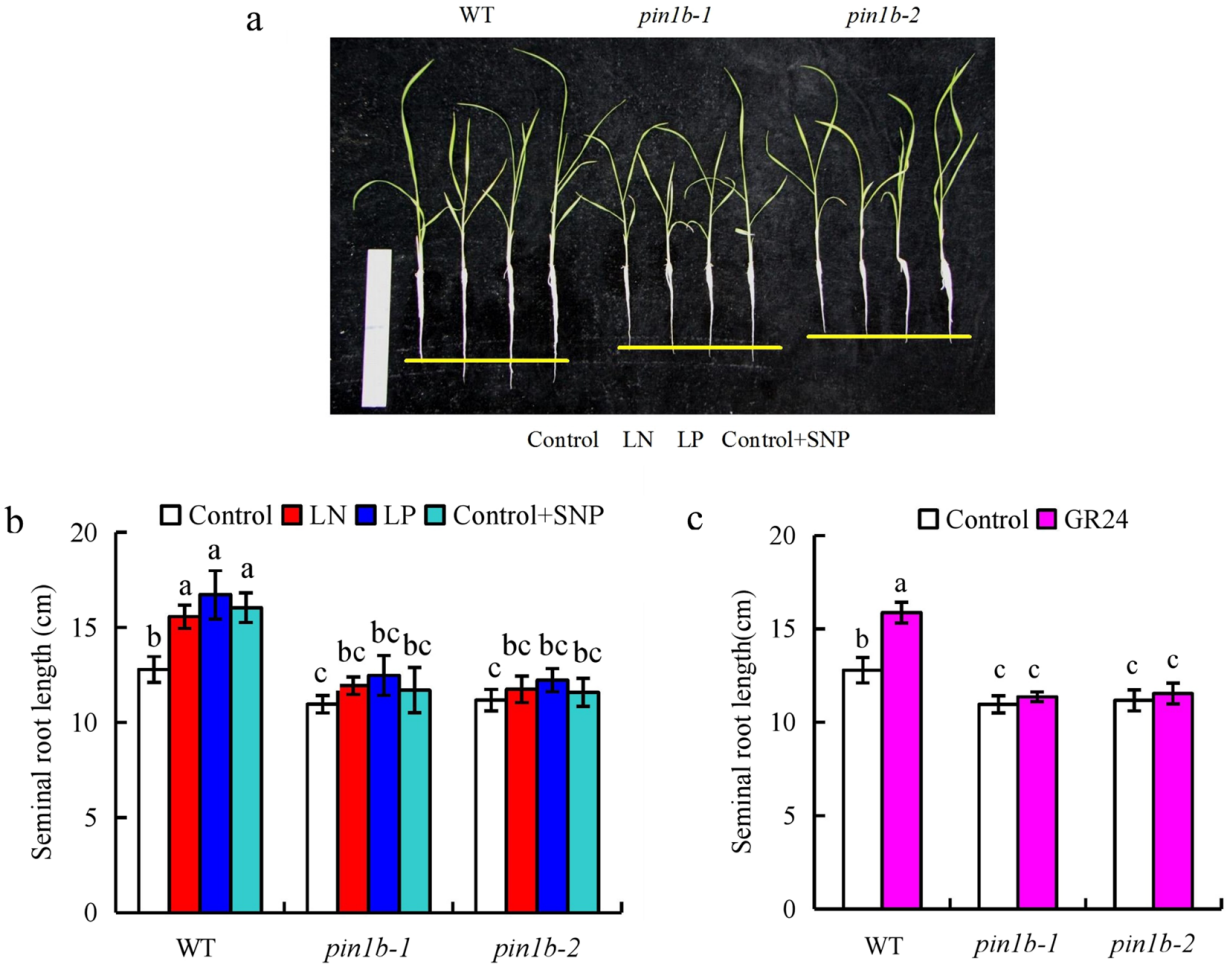
Seminal root length in the wild type (WT, Dongjin) and *ospin1b* mutants. Seedlings were grown in hydroponic medium containing normal nutrition (control; 2.5 mM N, 300 μM ), low N, or low P (LN, 0.02 mM; LP, 2 µM P), or subjected to treatment with sodium nitroprusside (SNP, 10 µM) or GR24 (analog of SLs, 2.5 µM) for 14 days. (**a**), Morphology and (**b**,**c**) seminal root lengths of rice plants. Data are means ± SE of eight replicates (**b**,**c**) and bars with different letters indicate significant differences at p < 0.05, as determined by ANOVA followed by the LSD test.

[^3^H]IAA transport and auxin concentration in the root tip, as well as the seminal root length of *ospin1b* mutants, were unaffected by LN, LP, SNP, and GR24 (Supplementary Fig. S5). Compared with WT plants, shorter seminal root length was recorded in two *ospin1b* mutants under different treatments. Compared with the control, the length of WT was increased under LN, LP, SNP, and GR24 treatments. However, the length of *ospin1b* mutants was not affected under control, LN, LP, SNP, or GR24 treatments (Fig. 6). These findings confirmed that *OsPIN1b* is involved in LN and LP in NO- and SL-mediated regulation of seminal root length.

### LN and LP conditions, and SNP and GR24 application, do not influence *pCYCB1;1::GUS* expression in the *ospin1b* mutants

**Figure 7:**
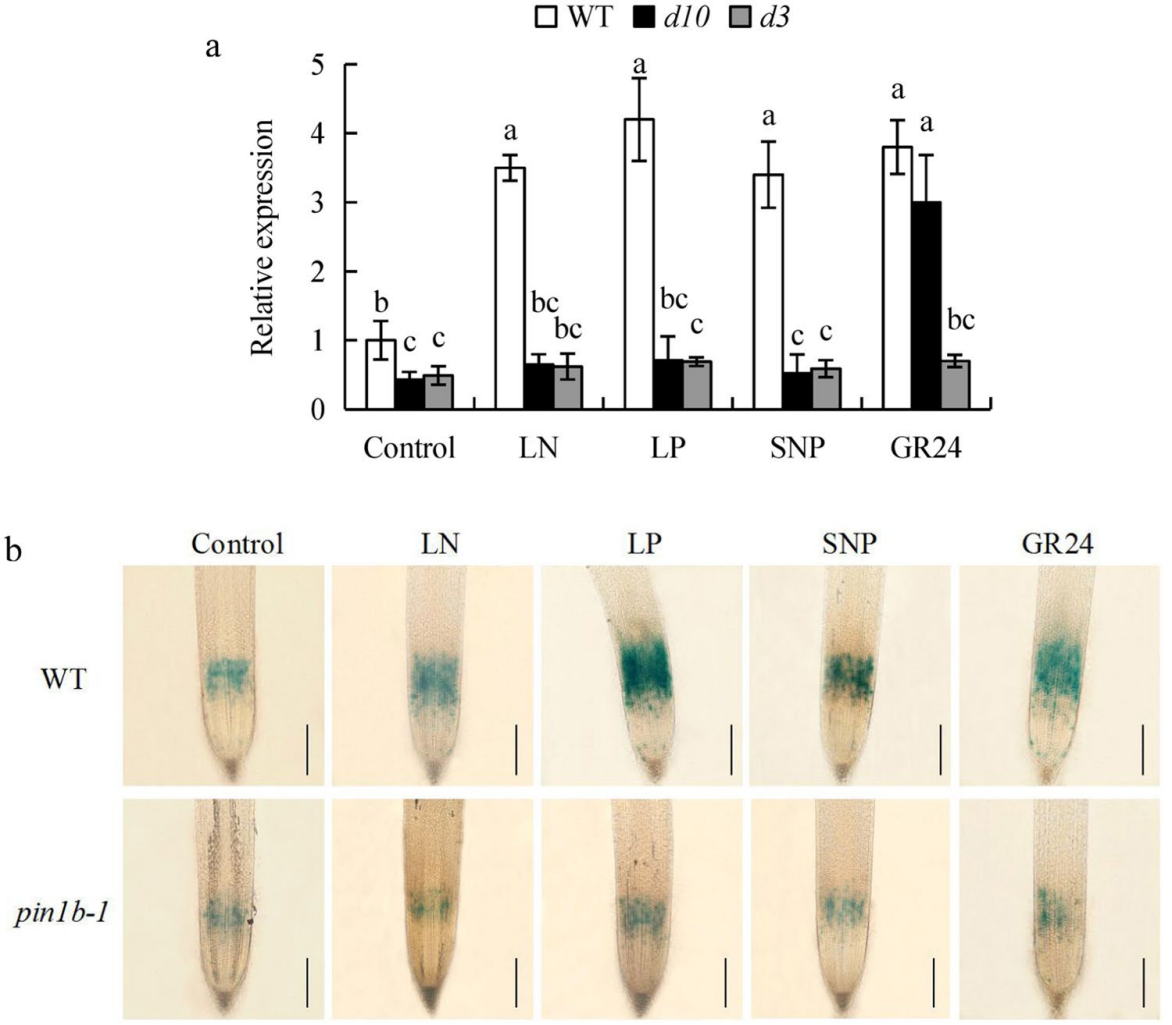
*OsCYCB1;1* gene and *pCYCB1;1::GUS* expression in rice seedlings. Seedlings were grown in hydroponic medium containing normal nutrition (control; 2.5 mM N, 300 μM P), low N, or low P (LN, 0.02 mM; LP, 2 µM), or subjected to treatment with sodium nitroprusside (SNP, 10 µM) or GR24 (analog of SLs, 2.5 µM) for 14 days. (**a**) The expression of *OsCYCB1;1* gene in WT, *d10* and *d3* mutants. (**b**) *pCYCB1;1::GUS* expression in WT and *ospin1b-1* mutant. Cell cycle activity of the root meristem, as monitored by the *pCYCB1;1::GUS* reporter, is shown. Bar = 500 μm. Data are means ± SE of three replicates (**a**) and bars with different letters indicate significant differences at p < 0.05, as determined by ANOVA followed by the LSD test.

NO influences root elongation primarily by regulating root meristem activity^43^. In this study, the relative expression of *OsCYCB1;1* was significantly decreased in *d10* and *d3* mutants relative to WT under control, LN, LP, and SNP treatments. However, application of GR24 markedly increased the levels of *OsCYCB1;1* in *d10*, but not in *d3* mutants, to the same extent as in WT (Fig. 7a).

*ospin1b-1* transgenic plants expressing the *pCYCB1;1::GUS* construct were used to assess cell cycle activity in the root meristem (Fig. 7b). Compared to control conditions, *pCYCB1;1::GUS* expression in WT plants was greater under LN and LP conditions and following application of SNP and GR24. However, no significant change in *pCYCB1;1::GUS* expression was recorded in the root tip of the *ospin1b-1* mutant. These findings suggested that *OsPIN1b* is involved in the role of NO and SLs in LN- and LP-induced root apical meristem activity.

## Discussion

### Strigolactones and strigolactone signaling are required for NO-mediated suppression of auxin transport to root apex

Auxin acts as a signaling intermediate in the response of root architecture to low phosphate supply^45^. An auxin transport-independent pathway is involved in the changes in primary roots induced by phosphate stress in *Arabidopsis*^3,6,7,46,47^. Studies in *Arabidopsis* have provided insight into the crosstalk between auxin and the root elongation response to P deficiency^9,48^. Moreover, auxin participates in the regulation of root elongation under N- and P-deficient conditions in rice^12^.

SLs, NO, and auxin transport are closely linked in the regulation of primary root elongation^12,30^. NO functions as a signaling molecule in the inhibition of auxin transport to the root apex and restrains primary root elongation in *Arabidopsis*^30^. In addition to NO, SLs may act as modulators of auxin transport to regulate root elongation^12,32^. In *Arabidopsis*, SLs modulate local auxin levels in a manner dependent on the auxin status of the plant^32^. In rice, the application of SLs markedly reduces auxin transport under N- and P-deficient conditions, which in turn increases seminal root length^12^.

In this study, compared with those in the control conditions, *pDR5::GUS* intensity, [^3^H]IAA activity, and IAA concentration in root tips were significantly reduced under LP and LN conditions (Supplementary Figs S1b,e and 2a), consistent with a previous report^12^. The application of SNP and GR24 under normal nutrition conditions and the application of cPTIO under low-nutrient conditions decreased and increased *pDR5::GUS* intensity and [^3^H]IAA activity in root tips, respectively, to levels similar to those under low-nutrient or control conditions (Supplementary Figs S1c,e and 2a). Furthermore, the application of NAA or NPA reversed the effect of SNP and cPTIO on seminal root length and *pDR5::GUS* expression (Supplementary Fig. S1). These results suggested that NO and SLs participate in LN- and LP-induced rice seminal root elongation by downregulating polar auxin transport from the shoot to the root tip.

We reported previously that SLs participate in LN- and LP-modulated seminal root elongation by inhibiting auxin transport^12^. In this study, *pDR5::GUS* expression, [^3^H]IAA transport, and *OsPIN* family expression were unaffected by SNP application, and *OsPIN1b* expression was unaffected by LN, LP, LN + cPTIO, and LP + cPTIO application, in the root tips of the *d10* mutants (Figs 3 and 4; Supplementary Fig. S2). These results suggested that SL synthesis and signaling are required for the effect of NO on polar auxin transport from the shoot to root tip in rice.

### *OsPIN1b* is involved in NO- and SL-induced root elongation in response to low N and P

The localisation of PINs in rice roots was reported by Wang *et al*.^44^: among rice 10 *PIN* genes, only *OsPIN1b* was expressed in the root cap; *OsPIN1b/1c/5a/5b* were expressed in the meristem; *OsPIN1b/1c/9* were detected mainly in stele; *OsPIN1a/1c/9/10a/10b* were found in the LR cap region and *OsPIN1c* was also expressed in lateral root primordia (LRP); and *OsPIN9* has higher activity in LRP and stele^44^. AtPIN1 was the first putative auxin efflux carrier to be characterised in *Arabidopsis*^49^. Subsequently, OsPIN1, ZmPIN1a, and ZmPIN1b were characterised in rice^22^ and maize^50^. Phylogenetic analysis showed that AtPIN1 is a highly conserved gene that may play a key role in polar auxin transport^51^. Gene structure analysis showed that rice has four *OsPIN1* genes (*OsPIN1a/1b/1c/1d*), the distribution of each of which is similar to that of AtPIN1^44^. AtPIN1 and OsPIN1b regulate root growth in different plant species^22,30^. Mutation of *atpin1* decreased the length of primary root by influencing the root tip cell meristem^30^. *ospin1b* RNAi transgenic plants have fewer adventitious roots, but a similar root length, compared to WT plants^22^. However, we found that [^3^H]IAA transport and IAA concentration in root tips of *ospin1b* were unaffected by LN, LP, SNP, and GR24 (Supplementary Fig. S5), and seminal root length was shorter than that of WT plants under LN and LP conditions, and following SNP and GR24 application, which is inconsistent with a previous report^22^. This is likely due to our use of knockout T-DNA mutants while their RNAi transgenic plants are knockdown of *OsPIN1b*.

The influence of NO and SLs on auxin transport involves regulation of PIN proteins^30,32^. For example, the transcriptional and translational levels of AtPIN1 in root tip were reduced by the application of NO donors^30^. Strigolactones act by increasing the rate of AtPIN1 removal from the plasma membrane in shoot, but not in root^52^. *OsPIN1a*, *1b*, 9, and *10a* expression in rice root was significantly decreased under LN and LP conditions, and following GR24 application^12^. In this study, *OsPIN1a-b*, *OsPIN2*, and *OsPIN10a-b* expression in the WT was significantly reduced by SNP treatment compared with that in the control (Fig. 2b). Therefore, the expression of only *OsPIN1a/1b/10a* was downregulated under LN and LP conditions and following application of SNP and GR24. The expression of *OsPIN2*/*10b* was decreased under SNP treatment but not under LN and LP conditions, suggesting *OsPIN2*/*10b* was not involved in NO-mediated regulation of auxin transport under LN and LP. *OsPIN1b* was strongly expressed in root cap and meristem in transgenic rice plants with OsPIN-promoter-driven GUS^44^. Similarly, *OsPIN1b* expression was strongest in rice tips; *OsPIN1b* expression was at least 12- and 1,000-fold higher than that of *OsPIN1a* and *OsPIN10a*, respectively, in rice root tips (Supplementary Fig. S3). These findings suggested that *OsPIN1b* is involved in the role of LN and LP in the regulation of auxin transport by SL and NO.

The seminal root of *ospin1b* mutants was shorter than that of WT plants and root development in *ospin1b* mutants was less affected by low-nutrient conditions and application of SNP and GR24 (Fig. 6). Moreover, abamine relieved the inhibitory effect of SNP on *OsPIN1b* expression (Fig. 5); *OsPIN1b* expression was significantly upregulated by cPTIO application under LN and LP conditions to a level similar to that in the *d10* mutant subjected to mock treatment (Fig. 5). These findings confirmed that *OsPIN1b* is involved in the role of LN and LP in SL- and NO-mediated elongation of seminal root by affecting root meristem activity (Fig. 7, Supplementary Fig. S6).

## Methods

### Plant materials and growth conditions

SL-deficient mutant (*d10*), SL signaling mutant (*d3*), and wild-type (WT, Shiokari) plants were provided by Shinjiro Yamaguchi of the Riken Plant Science Center. The T-DNA insertion *ospin1b* mutant lines (*ospinib-1* and *ospin1b-2*) and the WT (cv. Dongjin) plants were obtained from the Rice Functional Genomics Express Database (RiceGE, Pohang City, South Korea).

Plants were grown in a greenhouse under natural light at day/night temperatures of 30/18 °C. Seven-day-old seedlings of uniform size and vigour were transplanted into holes in a lid placed over the top of pots (four holes per lid and three seedlings per hole). Nutrient solutions varying from one-quarter to full strength were applied for 1 week, followed by full-strength nutrient solution for a further week. Pots receiving normal nutrition (control) were filled with 2.5 mM N (NH_4_NO_3_) and 300 µM P (KH_2_PO_4_), and those receiving N- and P-deficient nutrition were filled with 0.02 mM N (LN) and 2 µM P (LP). To exclude any potential effects of potassium (K^+^) on the treatments, the low-P treatment solutions were supplemented with K^+^ to the same levels as those under sufficient P conditions (300 µM) using K_2_SO_4_. The full chemical composition of the International Rice Research Institute (IRRI) nutrient solution was (mM): 0.35 K_2_SO_4_, 1.0 CaCl_2_, 1.0 MgSO_4_·7H_2_O, 0.5 Na_2_SiO_3_; and (µM), 20.0 Fe-EDTA, 9.0 MnCl_2_, 0.39 (NH_4_)_6_Mo_7_O_24_, 20.0 H_3_BO_3_, 0.77 ZnSO_4_, and 0.32 CuSO_4_ (pH 5.5). The nutrient solution was replaced with fresh solution daily. Each treatment consisted of four replicates arranged in a completely randomised design to avoid edge effects. In addition, all experiments included three independent biological replicates.

Pharmacological treatments comprising 10 µM SNP (NO donor), 80 µM cPTIO (a NO scavenger), 10 nM NAA (an analogue of IAA), 2.5 µM GR24 (an analogue of SLs), and 100 µM abamine (SL synthesis inhibitor) were applied to the hydroponic media. Since NAA is more stable than IAA in nutrient solution, NAA was used as the analogue of IAA in this study. Localised application of NNPA (a polar auxin transport inhibitor) was performed by dispensing diluted agar containing 20 µM NPA directly from a pipette across the root-shoot junction^23^.

### Measurement of root system architecture

Seminal roots were significantly longer than adventitious roots under our experimental conditions. Our preliminary experiment showed similar responses of seminal and adventitious roots to LN and LP treatments, and the number of adventitious roots did not change significantly under the different nutrient conditions during the experimental period^12,43^. Therefore, seminal roots were selected to study the effects of LN and LP on the rice root system. Seminal root lengths were measured using a ruler.

### Determination of IAA

IAA concentrations in root tips were determined as described previously^53^. The fresh weight of samples was first determined, followed immediately by freezing in liquid N_2_. Measurement of free IAA by high-performance liquid chromatography (HPLC) was carried out as described previously^53^. An IAA standard was obtained from Sigma-Aldrich (St. Louis, MO, USA).

### *pDR5::GUS* construct

To assess IAA distribution in rice plants, a *pDR5::GUS* construct was transformed into WT and *d10* and *d3* mutants using *Agrobacterium tumefaciens* (strain EHA105). The *pDR5::GUS* construct was provided by Professor Ping Wu’s group at Zhejiang University, Hangzhou, China. The samples used for IAA analysis were also used for histochemical GUS staining. The root tips were stained for GUS activity for 2 h at 37 °C. The stained tissues were photographed using an Olympus SZX2-ILLK stereomicroscope with a colour charge-coupled device (CCD) camera (Olympus, Tokyo, Japan)^12^.

### *pCYCB1;1::GUS* construct

*The pCYCB1;1::GUS* fusion construct was transformed into rice plants. The *pCYCB1; 1::GUS* construct was provided by Professor Chuanzao Mao’s group at Zhejiang University, Hangzhou, China. The root tips were used for histochemical GUS staining, and were photographed using an SZX2-ILLK microscope with a colour CCD camera.

### Measurement of NO in root tips

NO was imaged by diaminofluorescein-FM diacetate (DAF-FM DA) and epifluorescence microscopy. The root tips were loaded with 10 µM DAF-FM DA in 20 mM HEPES-NaOH buffer (pH 7.5). After incubating in the dark for 30 min, the root tips were washed three times in fresh buffer and immediately visualised using a stereomicroscope (Olympus MVX10) equipped with a colour CCD camera, with excitation and emission at 488 and 495–575 nm, respectively. Green fluorescence intensity was quantified using Photoshop software (Adobe Systems, San Jose, CA, USA)^54^. Data are presented as mean fluorescence intensities.

### [^3^H]IAA transport

Shoot-to-root auxin transport in intact plants was assayed as described previously^53^. Polar transport of [^3^H]IAA was assayed using eight replicate root samples. The [^3^H]IAA solution contained 0.5 µM [^3^H]IAA (20 Ci mmol-1) in 2% DMSO, 25 mM MES (pH 5.2), and 0.25% agar.

Shoot-to-root auxin transport in intact plants was monitored as follows. [^3^H]IAA solution (20 µL) was applied to the cut surface after removal of rice shoots 2 cm above the root-shoot junction. The root tips were incubated in 4 mL of scintillation solution for 18 h (overnight) in the dark, and then sampled and weighed. [^3^H]IAA radioactivity was detected using a multipurpose scintillation counter (LS6500; Beckman-Coulter, Fullerton, CA, USA).

### qRT-PCR

Total RNA was isolated from the root tips (0–0.5 cm) of rice seedlings. RNA extraction, reverse transcription, and qRT-PCR were performed as described previously^23^. Primer sets for the *OsPIN* genes are listed in Supplemental Table S1.

### Data analysis

Data were pooled for calculation of the means and standard errors (SE) and subjected to one-way analysis of variance (ANOVA) followed by a least significant difference (LSD) test at P < 0.05 to determine the significance of differences between treatments. All statistical evaluations were conducted using SPSS software (ver. 11.0; SPSS Inc., Chicago, IL, USA).

## Electronic supplementary material

Supplementary Dataset 1
