# Supplementary material for: OsPIN1b is Involved in Rice Seminal Root Elongation by Regulating Root Apical Meristem Activity in Response to Low Nitrogen and Phosphate
Source: Sci Rep. 2018 Aug 29;8:13014. doi: 10.1038/s41598-018-29784-x (PMC6115472; doi:10.1038/s41598-018-29784-x)
Supplement: Supplementary file 1 — Supplementary Dataset 1 [file 41598_2018_29784_MOESM1_ESM.doc]

***OsPIN1b* is Involved in Rice Seminal Root Elongation by Regulating Root Apical Meristem Activity in Response to Low-Nitrogen and -Phosphate**

**Huwei Sun****^1,2^,*****, Jinyuan Tao^1^,*, Yang Bi^1^, Mengmeng Hou^1^, Jiajing Lou^1^, Xinni Chen^1^, Xuhong Zhang^1^, Le Luo^1^, Xiaonan Xie^3^, Koichi Yoneyama^3^, Quanzhi Zhao^2^, Guohua Xu^1^, Yali Zhang^1, +^**

^1^State Key Laboratory of Crop Genetics and Germplasm Enhancement, and Key Laboratory of Plant Nutrition and Fertilization in Low-Middle Reaches of the Yangtze River, Ministry of Agriculture, Nanjing Agricultural University, Nanjing 210095, China

^2^College of Agronomy, Collaborative Innovation Center of Henan Grain Crops, Key Laboratory of Rice Biology in Henan Province, Henan Agricultural University, Zhengzhou 450002, China

^3^Center for Bioscience Research & Education, Utsunomiya University, 350 Mine-machi, Utsunomiya 321-8505, Japan

*These authors contributed to this work equally.

**Correspondence and requests for materials should be addressed to Y.L.Z.** [**(ylzhang@njau.edu.cn)**](mailto:(ylzhang@njau.edu.cn))

Table S1 The primers for qRT-PCR of *PIN and CYCB1;1* family genes.

| Gene | Primer sequence |
| --- | --- |
| *OsPIN1a* | 5'-TCATCTGGTCGCTCGTCTGC-3' |
|  | 5'-CGAACGTCGCCACCTTGTTC-3' |
| *OsPIN1b* | 5'-TGCACCCTAGCATTCTCAGCA-3' |
|  | 5'-CCCTCCTCCCAAATTCTACTT-3' |
| *OsPIN1c* | 5'-CCGTCAGGTTCCTCGTGGGT-3' |
|  | 5'-TCACGGCTGTGCTCAGAATG- 3' |
| *OsPIN2* | 5'-CAACACCTACTCCAGCCTC-3' |
|  | 5'-TGGACCAGTCAAGAACCTC-3' |
| *OsPIN5a* | 5'-GGGGCTGGTGCTAAAGTTCG-3' |
|  | 5'-TGAGGTAGGGCTGCCTGTATG-3' |
| *OsPIN5b* | 5'-GGGCAGCAGGAGAGGGTGATAG-3' |
|  | 5'-GAATCGGCAGAGAGATCAATGT-3' |
| *OsPIN8* | 5'-GTTCCACTATATGTAGCTATGATAC-3' |
|  | 5'-CAGTCAAACTTCTCTGCACAGC-3' |
| *OsPIN9* | 5'-GATACAAGATAGCGTCGTTCTC-3' |
|  | 5'-ATGATGTCTGCGTGGACCT-3' |
| *OsPIN10a* | 5'-GTTGGATTGAGATAGGCTGAGGAG-3' |
|  | 5'-ATGGCGACGAAGCGGTTGAT-3' |
| *OsPIN10b* | 5'-TCCGATGCAGGGTTAGGC-3' |
|  | 5'-AGGATGGTAGCGTGGAGGTT-3' |
| *OsCYCB1;1* | 5'-CACGTCGACTATAGACTAAGCCATTGAGGCGTAT -3' |
|  | 5'-AAAGGTACCAGAGCTGATCTCGATGACATGCTCGG-3' |
| *OsACTIN* | 5'-CAACACCCCTGCTATGTACG-3' |
|  | 5'-CATCACCAGAGTCCAACACAA-3' |

c

a

d

b

e

h

f

**Fig. S1.** The accumulation of nitric oxide (NO) and histochemical localization of *pDR5::GUS* activity and IAA concentration in root tip and the length of seminal roots in wild-type (WT) rice seedlings (Shiokari). Seedlings were grown in hydroponic medium containing normal nutrition (Control; 2.5 mM N, 300 μM P), low-N and -P (LN, 0.02 mM; LP, 2 µM P) in addition to application of Sodium nitroprusside (SNP, 10 µM), 2-(4-Carboxyphenyl)-4,4,5,5- tetramethylimidazoline-1-oxyl-3-oxie (cPTIO, 80 µM) and GR24 (analog of SLs, 2.5 µM) for 14 days. (a), NO production shown as green fluorescence in the root tips; (b,f), *pDR5::GUS*, a specific reporter that contains seven repeats of a highly active synthetic auxin response element and can reflect the in vivo auxin level. Plants were stained for *GUS* activity for 2 h at 37°C. (c), NO production in the root tips expressed as fluorescence intensity. (d, h), Seminal root length. (e) IAA concentration in root tip; Bar=1mm. Data are means ± SE of eight replicates and bars with different letters indicate significant differences at p<0.05, as determined by ANOVA followed by the LSD test.

**Fig. S2** qRT-PCR analysis of *PIN* family genes of root in wild-type (Shiokari) and *d10* mutant rice seedlings. Seedlings were grown in hydroponic medium containing normal nutrition (Control; 2.5 mM N, 300 μM P) in addition to application of Sodium nitroprusside (SNP, 10 µM) for 14 days. Data are means ± SE of eight replicates and bars with different letters indicate significant differences at p<0.05 in the same gene, as determined by ANOVA followed by the LSD test.

**Fig. S3.** qRT-PCR analysis of *PIN* family genes of root tips in the wild-type (WT) rice seedlings (Shiokari). Seedlings were grown in hydroponic medium containing normal nutrition (Control; 2.5 mM N, 300 μM P) for 14 days. Data are means ± SE from three replicates.

a

b

c

**Fig. S4** Identification of T-DNA insertion *ospin1b* mutants. (a), Gene structure of *OsPIN1b* and the T-DNA insertion sites. The exons and introns are indicated by white boxes and lines, respectively; the promoter and URT regions are indicated by striated box and bold boxes, respectively. (b) Identification of homozygous T-DNA insertion mutants by two rounds of RT-PCR. (c) qRT-PCR analysis for the expression of *OsPIN1b* in roots of WT and *ospin1b-1* (PEG_2A-08763.L) and *ospin1b-2* (PEG_3A-04335.R) mutants. Data are means ± SE from three replicates and bars with different letters indicate significant differences at p<0.05, as determined by ANOVA followed by the LSD test.

b

a

**Fig. S5** [^3^H]IAA transport and IAA concentration in root tips of *ospin1b-1* mutant.

Seedlings were grown in hydroponic medium containing normal nutrition (Control; 2.5 mM N, 300 μM P), low-N and -P (LN, 0.02 mM; LP, 2 µM P) in addition to application of Sodium nitroprusside (SNP, 10 µM) and GR24 (analog of SLs, 2.5 µM) for 14 days. (a), [^3^H]IAA transport. (b), IAA concentration in root tips. Data are means ± SE from eight replicates and bars with different letters indicate significant differences at p<0.05, as determined by ANOVA followed by the LSD test.

**Fig. S6** Schematic model of the interaction among auxin, strigolactones and nitric oxide (NO) in regulating root elongation under low N and P in rice. NR (Nitrate Reductase ) and NOS (Nitric Oxide Synthase); Low N (0.02 mM) and Low P (2 µM).
